# Analysis of Differential miRNA Expression in Primary Tumor and Stroma of Colorectal Cancer Patients

**DOI:** 10.1155/2014/840921

**Published:** 2014-07-10

**Authors:** Giuseppina Della Vittoria Scarpati, Enrica Calura, Mariacristina Di Marino, Chiara Romualdi, Luca Beltrame, Umberto Malapelle, Giancarlo Troncone, Alfonso De Stefano, Stefano Pepe, Sabino De Placido, Maurizio D'Incalci, Sergio Marchini, Chiara Carlomagno

**Affiliations:** ^1^Department of Clinical Medicine and Surgery, University Federico II, Via Sergio Pansini 5, 80131 Napoli, Italy; ^2^Department of Biology, University of Padova, Via 8 Febbraio 2, 35122 Padova, Italy; ^3^Department of Oncology, Istituto di Ricerche Farmacologiche Mario Negri, Via La Masa 19, 20156 Milano, Italy; ^4^Department of Public Health, University Federico II, Via Sergio Pansini 5, 80131 Napoli, Italy; ^5^Department of Medicine, University of Salerno, Via Salvatore Allende 43, 84084 Baronissi, Italy

## Abstract

Microarray technology was used to profile miRNA expression in primary tumor and stromal tissue from paraffin embedded material of 51 patients with colorectal cancer. 26 miRNAs resulted differentially expressed with at least 2-fold change in tumor tissue with respect to stroma (16 more expressed in the tumor and 10 more expressed in the stroma). 10/26 were confirmed as differentially expressed at qRTPCR: miR-200c-3p, miR-141-3p, miR-200b-3p, miR-200a-3p, miR-1246, miR-92a-3p, miR-194-5p, miR-192-5p, miR-3651-5p, and miR-574-3p. No significant association was found between miRNA expressions and stage at diagnosis, site of primary tumor, first site of metastasis, progression-free, or overall survival.

## 1. Introduction

MicroRNAs (miRNAs) are small noncoding RNA molecules of 18–25 nucleotides in length, which regulate several cellular mechanisms such as differentiation, proliferation, survival, and apoptosis [1]. They function as negative posttranscriptional regulators of protein expression, by interacting with specific miRNA and their degradation. Depending on their up- or downregulation, miRNAs can act as either tumour suppressors or oncogenes in the tumorigenesis process. For this reason, many studies have investigated the role of miRNAs in the development of cancer [2, 3] and their capability to influence prognosis [4–6] and response to treatments. Several studies have focused on miRNAs expression profiling in colorectal cancer [7–10], discovering specific expression profiles in different steps of cancerogenesis [11] and in different stages of invasive cancer [12–15]. Recently, research has been focused on gene expression analysis in tumoral stroma that interacts with cancer tissues directly or indirectly through cytokines, creating a habitat suitable for the cancer development [16]. Some reports found out a different expression of oncogenes and tumor suppressor genes in stromal tissues, such as downregulation of PTEN and p53, which play a key role in breast cancer progression, or the ablation of TGFBR2 in fibroblasts that can lead to carcinogenesis* in vivo* [17, 18]. Furthermore, a number of studies revealed that gene expression status of cancer stroma may be related to prognosis as well as clinic and pathologic factors, revealing that the aggressiveness of cancer could be defined by gene expression patterns in stromal tissue. Fukino et al. [19] have shown that cancer-specific loss of heterozygosity (LOH) of some alleles or allelic imbalance in stromal cells, rather than those presenting in epithelial cells, is more likely to correlate with pathologic characteristics of the disease. These findings suggest that cancer stromal tissues are actively involved in cancer progression. Gene expression in cancer stroma could be also regulated by miRNAs expressed. Nishida et al. [20] investigated miRNA expression profiling in stroma of colorectal cancer tissue and normal epithelium, and they identified two clusters, miR-17-92a and miR-106b-25 cluster, that were upregulated in cancer stromal tissues, as compared with normal stroma. Gene expression analyses of the same stromal tissue samples showed that putative targets of these miRNAs, identified by Target Scan, such as TGFBR2, SMAD2, and BMP family genes, were significantly downregulated in cancer stromal tissue. They also investigated whether miRNA expression status in stromal tissue could influence the clinicopathologic factors and found that high expression of miR-25 and miR-92a in stromal tissues was associated with vascular invasion and liver metastases.

The aim of present study is to typify, by microarray, the differential expression profile of miRNAs between tumor tissue and stroma, which have been selected using the laser capture microdissection technique, in a group of patients affected by colorectal cancer.

## 2. Materials and Methods

### 2.1. Patients

Fifty-seven patients have been selected for the present analysis, with the following criteria: histologic diagnosis of colorectal cancer; being treated at the Unit of Medical Oncology of the University Federico II for metastatic disease; availability of a cancer-containing block from the primary tumour. All the patients received chemotherapy plus bevacizumab as first-line treatment, and were staged with whole body CT-scan before treatment start, and disease evaluation was scheduled every two months by repeating a WB CT-scan, and response was evaluated according to the RECIST criteria. Detailed characteristics of the patients are described in Table 1.

### 2.2. Tissue Microdissection

**Figure 1:**
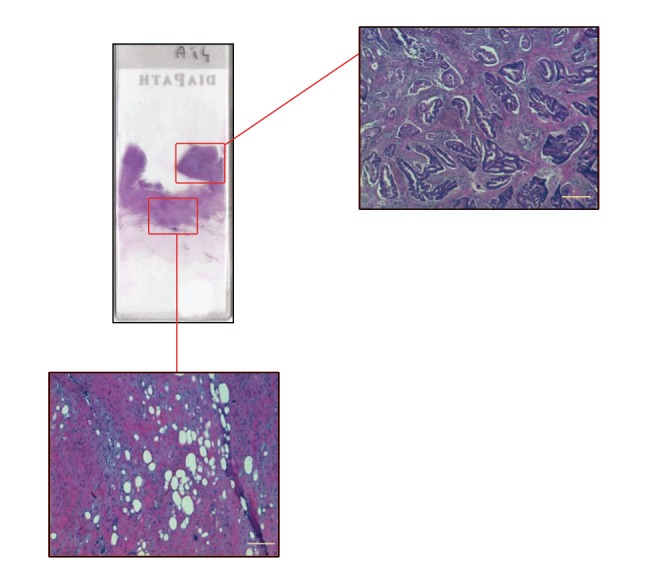
Graphic representation of the microdissection procedure carried out to selectively distinguish between tumor tissue and surrounding stroma (magnification bar = 50 microns).

A formalin-fixed paraffin-embedded (FFPE) block relative to the surgical specimen or to the diagnostic biopsy of the primary colorectal tumor was retrieved from the pathology archive. Alongside the corresponding hematoxylin and eosin (H&E) stained slide, tumour and stromal tissue areas were selectively distinguished with a permanent marker by a pathologist (GT) to guide microdissection (Figure 1). This procedure consisted of melting the paraffin block at 65°C for one hour to liberate the tissue from the surrounding paraffin and then to facilitate the separation of the tumor tissue from the stromal area with a scalpel, followed by reconstruction in two different paraffin blocks, one with the tumor area and a second block with the stromal tissue. From each of these blocks, depending on tissue sizes, four or five 6 *μ*m thick serial sections were cut and collected in Eppendorf tubes.

### 2.3. RNA Purification

RNA enriched in miRNAs fraction was purified separately from the paraffin-embedded tissues derived from tumor tissue and stroma through robotic workstation (QIAcube, Qiagen) by using miRNeasy FFPE kit isolation system following manufacturer's protocols (Qiagen). Total RNA concentration and proteins contamination were determined by Nanodrop spectrophotometer (Nanodrop Technologies, Ambion).

### 2.4. Microarray Experiments and Data Analysis

Array experiments were performed using standardized procedures, as previously described [21]. Briefly, 100 ng was CY5-labelled and hybridized with a miRNA labeling and hybridization kit according to manufacturer's instructions (Agilent Technologies, Palo Alto, CA, USA). We used commercially available G13 human miRNA microarray kit (Agilent Technologies), which consists of 60 K features printed in 8-plex format (8 × 15 array) that can detect all known miRNAs sourced from the Sanger miRBASE public database, release 19. Arrays were washed and scanned with laser confocal scanner (G2565BA) according to manufacturer's instructions. miRNA microarray underwent standard posthybridization processing and the intensities of fluorescence were calculated by features extraction software, version 11 (Agilent Technologies). Data were used for downstream analysis only for those samples with an additive error being equal to or below 5, which is an indirect measure 11 of quality of hybridized RNA and hybridization steps.

### 2.5. miRNA Microarray Analysis

Raw microarray data were preprocessed and normalized using the robust multichip average algorithm (RMA) [22, 23], keeping only probes detected in at least 60% of the samples. Differentially expressed miRNAs were calculated using linear model [24, 25], comparing each tumor sample with its corresponding stroma. miRNAs were called significant if their false discovery rate (FDR) was equal to 0.05. Supervised and unsupervised clustering were carried out on the differentially expressed miRNA using multiscale bootstrap resampling [26] over 1000 iterations, using Pearson's correlation coefficient as distance metric and average linkage. In accordance with the MIAME guidelines, array data have been submitted to the Array Express database (E-MTAB-2479).

### 2.6. Signature Validation Using qRT-PCR

miRNA expression levels were validated by quantitative reverse transcription polymerase chain reaction (qRT-PCR), using Sybr Green protocols and commercially available assays (Qiagen) on an Applied Biosystems 7900HT instrument. Experiments were run in triplicate, using 384-well reaction plates in an automatic liquid handling station (epMotion 5075LH; Eppendorf). Analysis was conducted as previously described [21], using 4 independent housekeeping genes (SNORD 72, SNORD 95, RNU 6B, and RNU 5A). Cycles' threshold for selected genes was in line with those obtained in the past for snap frozen tumor tissue in our experimental condition, which is an indirect measure of the good quality of RNA used for the analysis. To verify mean differences among groups, normalized PCR data were analyzed using Wilcoxon rank test using GraphPad Prism version 6.03 for Windows (GraphPad Software, La Jolla, California, USA, http://www.graphpad.com/). A two-sided *P* value < 0.05 was considered statistically significant.

### 2.7. Variables

The expression of miRNAs in the primary tumour was correlated with the following variables: (1) miRNA expression in the stroma; (2) stage at diagnosis (TNM II-III versus TNM IV); (3) first site of metastases (liver only versus lung only); (4) site of primary tumour (right colon versus left colon versus rectum).

Moreover, miRNAs' level of expression was correlated with the progression-free survival (PFS), defined as the time elapsed from the date of the first cycle of therapy for the metastatic disease and the date of documented progression, and the overall survival (OS), defined as the interval from date of the first cycle of therapy for the metastatic disease and the date of death for any cause or the last follow-up visit.

### 2.8. Survival Analysis

The potential prognostic role of miRNAs level of expression in the primary tumor was investigated. Survival of patient subgroups defined by their different levels of miRNA expression was tested using Kaplan-Meier method [27] and significance was assessed with two-sided long-rank statistics. Patients known to be alive at the time of analysis were censored at their last available contact date. The Cox proportional hazards model was used to determine the risk ratios and *P* value for multivariate analyses. All the statistical analyses were performed using the R programming language (R version 2.14) and the BioConductor software suite (version 2.9).

## 3. Results

### 3.1. miRNA Profiling

To identify the entire repertoire of miRNAs differentially expressed between primary tumor and adjacent stroma, in patients affected by metastatic colorectal cancer, a cohort of 51 matched FFPE tumor-stroma samples were profiled by microarray technology. Quality control criteria selected a signature of 321 miRNAs worthy of downstream analysis.

miRNA expression profiles were used to investigate two main issues: (i) the possible association among miRNA expression profiles of tumour samples and clinical variable (such as stage, site of primary tumor and metastasis, and survival) and (ii) molecular differences between tumor and stroma samples.

Regarding the first aim, we did not find any miRNAs in the tumor differentially expressed according to stage at diagnosis (limited versus metastatic), primary tumor sites (three categories: (1) right colon + transverse colon; (2) left colon + sigma; (3) rectum) and first metastasis site (liver versus lung). Furthermore we did not find any miRNAs significantly associated with OS and PFS.

Then we proceed to analyze differences between tumor and stroma samples. To identify groups of samples with specific patterns of miRNAs' expression, firstly we performed unsupervised hierarchical cluster analysis on all 321 miRNAs which allowed us to investigate similarities or differences between the tumor and stromal samples. To ensure that no biases were present in the data, we used a resampling approach by randomly permuting samples and microRNAs 1000 times, calculating a confidence level on the results of the clustering. Data showed with level of confidence 95% (see Supplementary Figure 1 in Supplementary Material available online at http://dx.doi.org/10.1155/2014/840921) two heterogeneous clusters, with no clear grouping of the samples.

Secondly, we decided to focus our analysis only on those miRNAs differentially expressed between tumor and stroma. With a *P* value < 0.05, we identified 134 miRNAs that were differentially expressed between the tumor tissue and the stroma across all samples. Of these, 87 were overexpressed in the tumor as compared to the matched stroma slices, while 47 were more expressed in the stroma as compared to the matched tumor slices (Supplementary Table 1). Among the differentially expressed miRNAs, we selected those which changed at least 2-fold between tumor and stroma, obtaining 26 differentially expressed miRNAs (16 more expressed in the tumor and 10 more expressed in the stroma) (Table 2). Using mirTarBase [28], for each differentially expressed miRNA we select the number of validated targets (with reported assay or western blot); then, on this list, for each miRNA, we perform a pathway enrichment analysis using GraphiteWeb [29] and DAVID web tools [30]. Finally we used the human miRNA Disease Database (HMDD v. 2.0, 31) to include (if present) recent publications (with their brief description) assessing the involvement of these miRNAs on colorectal cancer. All this information is reported in Supplementary Table 2. To go more in detail in data analysis, we searched the list of miRNAs reported in Table 2 for miRNA families. Members of the miR-8 family (miR-200c-3p, mir-141-3p, and miR-200b-3p), as well as members of the miR-192/194, resulted the most upregulated miRNAs in the tumor compared to the matched stroma tissue, while the top downregulated miRNAs included hsa-miR-195-5p, hsa-miR-143-3p, and hsa-miR-133b.

**Figure 2:**
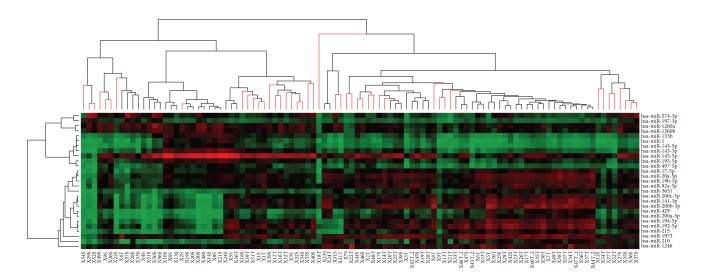
Bootstrap clustering analysis on tumor and stromal samples on the 26 differentially expressed microRNAs. Each row represents a single microRNA, and each column represents a sample (T, tumor; S, stroma). Red colors on the heat map indicate expression levels higher than the median, while green colors show expression levels lower than the median. The red branches on the dendrogram indicate an approximately unbiased (AU) confidence greater than 95%.

Third, we performed a supervised cluster analysis by setting a priori the two groups, stroma and tumor. Boostrap analysis reported in Figure 2, revealed with a level of 95% of clustering confidence, two main groups: the first consisting of stromal samples and the second mainly including tumor samples, with clear differences in the expression of subsets of miRNAs. In order to further classify subgroups of samples and microRNAs, we also clustered samples according to the relative change of expression (fold change) between tumor and stroma (Supplementary Figure 2).

### 3.2. miRNA Validation by qRT-PCR

**Figure 3:**
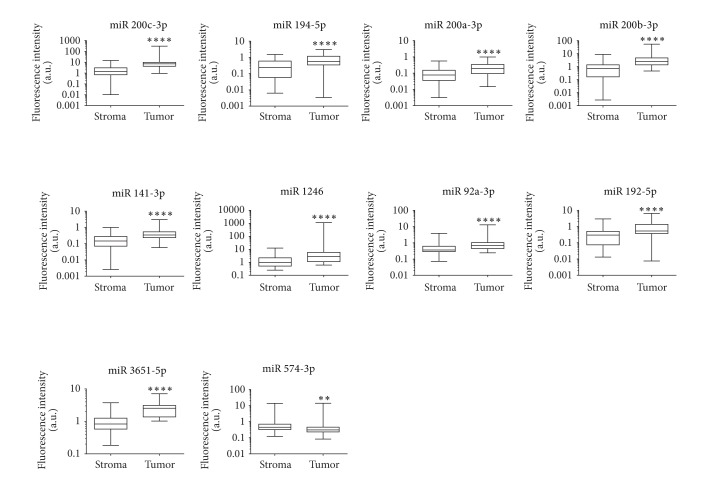
RT-PCR validation of DE miRNA in tumor versus stroma matched samples. Box plots show the median expression levels of the 10 miRNAs confirmed as upregulated in tumor samples, estimated in terms of normalized fluorescence intensity. Significant differences at Wilcoxon Rank test with a percentile range of 2.5–97.5 are reported (****P value < 0.0001).

To assess the reproducibility and robustness of the miRNA signature identified by array analysis, we measured by qRT-PCR the expression of 13 out of 26 top ranked miRNAs differentially expressed in the tumor and the stroma (Supplementary Table 3). The Wilcoxon rank test confirmed the difference in expression level for 10 of the 13 selected miRNAs. Box plots reported in Figure 3 show the median distribution levels of normalized fluorescence intensity and the IQ-R of the 10 upregulated or downregulated miRNAs obtained by qRT-PCR analysis. Of note, 9 out of the 10 miRNAs analyzed are more expressed in the tumor than in the stroma, whereas 1 is downregulated.

## 4. Discussion

In the present study, we analyzed by microarray the expression level of miRNAs in cancer stroma and tumor cells of primary colorectal carcinoma, and we found that 26 miRNAs are differentially expressed between the tumor tissue and the stroma: 16 have higher level of expression in the tumor than in the stroma, and 10 showed higher level in the stroma than the tumor. It is worth noting that most of these miRNAs have several validated target genes whose involvement regards different cancer pathways, p53 signalling, cell cycle control, and other pathways involved in cancer development and progression (Supplementary Table 2). Moreover, some of them have been already found to be involved in colorectal cancer (Supplementary Table 2).

Nine out of these 26 miRNAs (hsa-miR-200c-3p, hsa-miR-200a-3p hsa-miR-200b-3p hsa-miR-194-5p hsa-miR-141-3p, hsa-miR-92a-3p, hsa-miR-192-5p, hsa-miR-3651-5p, and hsa-miR-1246) were confirmed by qRT-PCR to be more expressed in tumor with respect to the corresponding stroma. Three of the miRNAs more expressed in the tumor are part of the “miR-200 family,” which is known to have an important regulatory activity in the phenomenon called epithelial-mesenchymal transition (EMT). According to the EMT, there is a molecular reprogramming of the cancer cells that lose their epithelial features and acquire mesenchymal characteristics and become able to move away from the primary tumor and give metastases, by invading stroma and vessels [31, 32].

Epithelial-mesenchymal (EMT) and mesenchymal-epithelial (MET) transitions occur in the development of human tumorigenesis and are part of the natural history of the process to adapt to the changing microenvironment. In this setting, the miR-200 family is recognized as a master regulator of the epithelial phenotype by targeting ZEB1 and ZEB2, two important transcriptional repressors of the cell adherence (E-cadherin) and polarity (CRB3 and LGL2) genes [31].

Gregory et al. showed that the members of the miR-200 family positively regulate E-cadherin expression, directly inhibiting ZEB1 and ZEB2, which have a central role in EMT, and this observation suggested that miR-200 can suppress migration and metastasis of cancer cells [33]. Understanding the regulation of EMT by miRNAs opens new avenues for the diagnosis and prognosis of tumors and identifies potential therapeutic targets that might help to negatively impact on metastasis dissemination and increasing patient survival.

Using the TargetScan program, in order to predict candidate miRNAs targeting ZEB genes, that is, miRNAs being able to bind the 3′UTR of ZEB2, they also include in this class of miRNAs miR-141 that resulted to be more expressed in the tumor than in the stroma in our series of cases. Therefore, on a closer view, four out of the ten miRNAs overexpressed in the tumor with respect to the adjacent stroma are potential regulator of the EMT, and they might control the tumor behavior.

High expression of miR-92a is associated with lymphatic and venous invasion and liver metastasis. In colon cancer, miR-17-92 sequence generates a single polycistronic transcript that gives rise to six different miRNAs, including miR-92-a. It is unclear what the target of miR-92-a is, but it has been suggested that it could target directly integrin-*α* and E-cadherin in epithelial cells. A recent research showed that miR-92-a downregulates a proapoptotic protein, known as Bim, and its overexpression is significantly related with lymph nodes metastases in colorectal cancer [34]. On this basis, the authors concluded that miR-92-a plays a central role in development and progression of colorectal cancer and in regulation of its aggressiveness.

miR-192 is mostly expressed in colon and liver and seems to be dependent on p53. It may be able to suppress cancerogenesis through p21 accumulation and cell cycle arrest [35]. Also miR-194 is p53-dependent and targets several genes involved in epithelial-mesenchymal transition and cancer metastasis. Overexpression of miR-194 in the mesenchymal-like liver cancer cell lines decreases N-cadherin expression and suppresses cell migration, invasion, and metastasis. For these reasons, miR-194 may have a potential role in maintaining the epithelial phenotypes of the cells and preventing EMT during cancer progression [36]. Though no data are nowadays available in our cohort of patients on mutational status of p53 gene, with the advent of massive parallel sequencing technology, it will be feasible early in the next feature to capture the mutational profile of the p53gene, integrating it with the miRNA landscape signature generated.

To our knowledge, there are no data about the role of miR-1246 and miR-574-3p in colorectal cancer development and progression. Interestingly, Zhang et al. [37] found that also the overexpression of miR-1246 may be induced by p53 and Piepoli et al. proposed that it is involved in MAPK signaling pathway. In addition, they found that the overexpression of miR-1246 reduced apoptosis, by interfering with DYRK1A (Down syndrome-associated protein kinase) [38], that is directly implicated in the resistance of cancer cells to proapoptotic signals and controls various pathways that regulate proliferation, migration, and inhibition of apoptosis, causing a very aggressive behavior of cancer [39]. In the matter of miR-574-3p, it was reported that it is a tumor suppressor miRNA and was found to be downexpressed in several types of cancer, such as gastric and bladder, and overexpressed in prostate cancer. By targeting mesoderm development candidate 1 (MESDC1), it is able to directly inhibit proliferation, migration, and invasion and to induce apoptosis [40]. The role of this miRNA in colorectal cancer has not been yet investigated. Similarly, no data are available about the role of miR-3651-5p in human cancer and our work seems to be the first that has found overexpression of this miRNA in colorectal cancer.

In conclusion, we found essential differences of expression in the two components of tumor bulk, neoplastic epithelium, and peritumoral stroma. In the neoplastic epithelium, profiling of miRNAs showed a simultaneous overexpression of favorable miRNAs, such as those that inhibit EMT, cell adhesion, and proliferation, and unfavorable miRNAs, those that can prevent apoptosis and promote EMT.

On basis of these findings, we can hypothesize that tumor aggressiveness may arise from the balance between negative and positive prognostic molecules expressed in tumor cells and microenvironment.

## Supplementary Material

Supplementary Figure 1. Bootstrap hierarchical clustering of normalized data passing the QC filter (321 microRNAs). Each column represents a sample (T, tumor; S, stroma). The red branches in the dendrogram indicate an approximately unbiased (AU) confidence level greater than 95%.
Supplementary Figure 2. Heatmap and bootstrap hierarchical clustering of differentially expressed microRNA in tumor versus stroma comparisons. Each row represents a single microRNA, and each column a matched tumor sample (T) versus stromal sample (S) comparison. Green colors indicate down-regulation (negative log fold change), while red colors indicate up-regulation (positive fold change). The red branches in the dendrograms indicate an approximately unbiased (AU) confidence greater than 95%.
Supplementary Table 1. Complete list of differentially expressed miRNA between tumor and matched stroma samples. ID, official miRNA name according to miRBASE version 19; logFC , log2 fold change of tumor expression versus stroma (negative for down-regulated miRNA and positive for up-regulated miRNA); p-value, the raw p-value from the statistical test; adjusted p-value, the p-value from the statistical test adjusted for multiple test comparisons (False Discovery Rate).
Supplementary Table 2: Number of validated targets, pathway enrichment of validated target and known association of differentially expressed miRNAs to colorectal cancer.
Supplementary Table 3. RT-PCR and microarray analysis of the 13 DE miRNA selected in tumor and matched stroma samples. All data are median distribution (IQ-range:2.5-97.5 percentile) of fluorescence intensity, normalized as described in the Methods section. R, the ratio of the median distribution of tumor to stroma samples measured by RT-PCR; P, the level of significance according to the Wilcoxon Rank test. Ra, the fold change (ratio) between tumor and stromal samples from the array analysis (in natural scale); q, the corrected p-value (q-value) from the array analysis.

## Figures and Tables

**Table 1:** Characteristics of the patients.

	*N*	%
Gender (M/F)	30/27	52.6/47.4
Age (median/range)	58 (31–81)	
Stage at diagnosis		
Early (TNM II or III)	24	42.1
Metastatic (TNM IV)	33	57.9
Site of primary		
Right colon	12	21.0
Rectum	16	28.1
Other segments	29	50.9
Number of metastatic sites		
1	45	78.9
≥2	12	21.1
Site of metastasis		
Liver only	28	49.1
Liver + others	10	17.5
Others	19	33.4
Chemotherapy		
FOLFIRI	47	82.5
FOLFOX/XELOX	7	12.3
FOLFOXIRI	3	5.2
Response to first-line therapy		
CR	6	10.5
PR	25	43.9
SD	22	38.6
PD	4	7.0

**Table 2:** List of miRNAs found differentially expressed between primary tumor and stroma.

ID	log⁡⁡FC	*P* value
hsa-miR-574-3p	−1.91158689	4.05*E* − 09
hsa-miR-145-5p	−1.74771641	1.09*E* − 04
hsa-miR-133b	−1.51232761	2.61*E* − 05
hsa-miR-143-3p	−1.43335307	2.08*E* − 04
hsa-miR-1	−1.34115539	9.46*E* − 05
hsa-miR-195-5p	−1.33523392	4.86*E* − 05
hsa-miR-143-5p	−1.23161021	5.98*E* − 05
hsa-miR-1260a	−1.1570189	2.19*E* − 07
hsa-miR-197-3p	−1.14830222	1.57*E* − 08
hsa-miR-1260b	−1.1083897	6.27*E* − 07
hsa-miR-200b-3p	2.40063273	8.10*E* − 08
hsa-miR-141-3p	2.22388859	8.10*E* − 08
hsa-miR-200c-3p	2.15323075	9.84*E* − 09
hsa-miR-192-5p	1.83444059	9.91*E* − 06
hsa-miR-194-5p	1.79583132	1.57*E* − 05
hsa-miR-200a-3p	1.67832962	3.74*E* − 07
hsa-miR-429	1.51819183	1.28*E* − 06
hsa-miR-215	1.507275	3.20*E* − 05
hsa-miR-3651	1.28775706	3.00*E* − 08
hsa-miR-210	1.19299762	1.18*E* − 04
hsa-miR-20a-5p	1.14909454	1.45*E* − 05
hsa-miR-1246	1.08433315	4.09*E* − 07
hsa-miR-17-5p	1.07625287	1.75*E* − 05
hsa-miR-92a-3p	1.06291954	1.15*E* − 06
hsa-miR-1973	1.04005254	1.82*E* − 05
hsa-miR-19b-3p	1.00428691	1.82*E* − 05

ID = official miRNA name according to miRBASE version 19; log⁡FC = log⁡2 fold change of tumor expression versus stroma (negative for downregulated miRNA and positive for upregulated miRNA); adjusted *P* value = the *P* value from the statistical test adjusted for multiple test comparisons (false discovery rate).
