# Supplementary material for: Analysis of Differential miRNA Expression in Primary Tumor and Stroma of Colorectal Cancer Patients
Source: Biomed Res Int. 2014 Jul 10;2014:840921. doi: 10.1155/2014/840921 (PMC4128171; doi:10.1155/2014/840921)
Supplement: Supplementary file 1 — Supplementary Figure 1. Bootstrap hierarchical clustering of normalized data passing the QC filter (321 microRNAs). Each column represents a sample (T, tumor; S, stroma). The red branches in the dendrogram indicate an approximately unbiased (AU) confidence level greater than 95%. Supplementary Figure 2. Heatmap and bootstrap hierarchical clustering of differentially expressed microRNA in tumor versus stroma comparisons. Each row represents a single microRNA, and each column a matched tumor sample (T) versus stromal sample (S) comparison. Green colors indicate down-regulation (negative log fold change), while red colors indicate up-regulation (positive fold change). The red branches in the dendrograms indicate an approximately unbiased (AU) confidence greater than 95%. Supplementary Table 1. Complete list of differentially expressed miRNA between tumor and matched stroma samples. ID, official miRNA name according to miRBASE version 19; logFC , log2 fold change of tumor expression versus stroma (negative for down-regulated miRNA and positive for up-regulated miRNA); p-value, the raw p-value from the statistical test; adjusted p-value, the p-value from the statistical test adjusted for multiple test comparisons (False Discovery Rate). Supplementary Table 2: Number of validated targets, pathway enrichment of validated target and known association of differentially expressed miRNAs to colorectal cancer. Supplementary Table 3. RT-PCR and microarray analysis of the 13 DE miRNA selected in tumor and matched stroma samples. All data are median distribution (IQ-range:2.5-97.5 percentile) of fluorescence intensity, normalized as described in the Methods section. R, the ratio of the median distribution of tumor to stroma samples measured by RT-PCR; P, the level of significance according to the Wilcoxon Rank test. Ra, the fold change (ratio) between tumor and stromal samples from the array analysis (in natural scale); q, the corrected p-value (q-value) from the array analysis. [file 840921.f1.zip › 840921.f1/840921.docx]

**SUPPLEMENTARY FIGURES AND TABLES LEGEND**

**Supplementary Figure 1. Bootstrap hierarchical clustering of normalized data passing the QC filter (321 microRNAs).**  Each column represents a sample (T, tumor; S, stroma). The red branches in the dendrogram indicate an approximately unbiased (AU) confidence level greater than 95%.

**Supplementary Figure 2. Heatmap and bootstrap hierarchical clustering of differentially expressed microRNA in tumor versus stroma comparisons.** Each row represents a single microRNA, and each column a matched tumor sample (T) versus stromal sample (S) comparison. Green colors indicate down-regulation (negative log fold change), while red colors indicate up-regulation (positive fold change). The red branches in the dendrograms indicate an approximately unbiased (AU) confidence greater than 95%.

**Supplementary Table 1. Complete list of differentially expressed miRNA between tumor and matched stroma samples.** ID, official miRNA name according to miRBASE version 19; logFC , log2 fold change of tumor expression versus stroma (negative for down-regulated miRNA and positive for up-regulated miRNA); p-value, the raw p-value from the statistical test; adjusted p-value, the p-value from the statistical test adjusted for multiple test comparisons (False Discovery Rate).

|  | **ID** | **logFC** | **pvalue** | **adjusted p-value** |
| --- | --- | --- | --- | --- |
|  | hsa-miR-574-3p | -1,9115869 | 1,26E-11 | 4,05E-09 |
|  | hsa-miR-200c-3p | 2,15323075 | 6,13E-11 | 9,84E-09 |
|  | hsa-miR-197-3p | -1,1483022 | 1,47E-10 | 1,57E-08 |
|  | hsa-miR-3651 | 1,28775706 | 3,74E-10 | 3,00E-08 |
|  | hsa-miR-141-3p | 2,22388859 | 1,44E-09 | 8,10E-08 |
|  | hsa-miR-200b-3p | 2,40063273 | 1,51E-09 | 8,10E-08 |
|  | hsa-miR-663b | 0,77767795 | 2,39E-09 | 1,10E-07 |
|  | hsa-miR-1260a | -1,1570189 | 5,46E-09 | 2,19E-07 |
|  | hsa-miR-200a-3p | 1,67832962 | 1,05E-08 | 3,74E-07 |
|  | hsa-miR-1246 | 1,08433315 | 1,38E-08 | 4,09E-07 |
|  | hsa-miR-1290 | 0,92604392 | 1,40E-08 | 4,09E-07 |
|  | hsa-miR-1260b | -1,1083897 | 2,35E-08 | 6,27E-07 |
|  | hsa-miR-92a-3p | 1,06291954 | 4,64E-08 | 1,15E-06 |
|  | hsa-miR-429 | 1,51819183 | 5,59E-08 | 1,28E-06 |
|  | hsa-miR-720 | -0,7269849 | 7,03E-08 | 1,50E-06 |
|  | hsa-miR-766-3p | -0,8696977 | 4,83E-07 | 9,70E-06 |
|  | hsa-miR-192-5p | 1,83444059 | 5,25E-07 | 9,91E-06 |
|  | hsa-miR-663a | 0,80827814 | 8,06E-07 | 1,44E-05 |
|  | hsa-miR-20a-5p | 1,14909454 | 8,80E-07 | 1,45E-05 |
|  | hsa-miR-3654 | 0,65584834 | 9,18E-07 | 1,45E-05 |
|  | hsa-miR-203 | 0,89749749 | 9,50E-07 | 1,45E-05 |
|  | hsa-miR-194-5p | 1,79583132 | 1,07E-06 | 1,57E-05 |
|  | hsa-miR-17-5p | 1,07625287 | 1,25E-06 | 1,75E-05 |
|  | hsa-miR-424-3p | 0,4557021 | 1,31E-06 | 1,75E-05 |
|  | hsa-miR-1973 | 1,04005254 | 1,47E-06 | 1,82E-05 |
|  | hsa-miR-19b-3p | 1,00428691 | 1,48E-06 | 1,82E-05 |
|  | hsa-miR-425-5p | 0,80064141 | 1,89E-06 | 2,25E-05 |
|  | hsa-miR-133b | -1,5123276 | 2,28E-06 | 2,61E-05 |
|  | hsa-miR-215 | 1,507275 | 2,89E-06 | 3,20E-05 |
|  | hsa-miR-195-5p | -1,3352339 | 4,54E-06 | 4,86E-05 |
|  | hsa-miR-664-5p | 0,52094262 | 5,27E-06 | 5,32E-05 |
|  | hsa-miR-365a-3p | -0,8131199 | 5,30E-06 | 5,32E-05 |
|  | hsa-miR-143-5p | -1,2316102 | 6,15E-06 | 5,98E-05 |
|  | hsa-miR-494 | 0,63509731 | 7,89E-06 | 7,45E-05 |
|  | hsa-miR-1 | -1,3411554 | 1,03E-05 | 9,46E-05 |
|  | hsa-miR-145-5p | -1,7477164 | 1,22E-05 | 1,09E-04 |
|  | hsa-miR-19a-3p | 0,94241736 | 1,33E-05 | 1,15E-04 |
|  | hsa-miR-210 | 1,19299762 | 1,40E-05 | 1,18E-04 |
|  | hsa-miR-133a | -0,8510897 | 1,99E-05 | 1,63E-04 |
|  | hsa-miR-497-5p | -0,9887135 | 2,03E-05 | 1,63E-04 |
|  | hsa-miR-143-3p | -1,4333531 | 2,66E-05 | 2,08E-04 |
|  | hsa-miR-3648 | 0,50113222 | 2,87E-05 | 2,20E-04 |
|  | hsa-miR-4324 | -0,5103669 | 3,05E-05 | 2,25E-04 |
|  | hsa-miR-130b-3p | 0,59123284 | 3,08E-05 | 2,25E-04 |
|  | ebv-miR-BART16 | -0,6430805 | 5,01E-05 | 3,58E-04 |
|  | hsa-miR-93-5p | 0,8305619 | 6,89E-05 | 4,71E-04 |
|  | hsa-miR-99b-5p | -0,8048456 | 6,90E-05 | 4,71E-04 |
|  | hsa-miR-500a-5p | 0,4459759 | 1,08E-04 | 7,22E-04 |
|  | hsa-miR-148a-3p | 0,93546999 | 1,23E-04 | 8,09E-04 |
|  | hsa-miR-3154 | 0,44026642 | 1,45E-04 | 9,29E-04 |
|  | hsa-miR-99a-5p | -0,9752663 | 1,81E-04 | 1,14E-03 |
|  | hsa-miR-3907 | -0,5207543 | 1,84E-04 | 1,14E-03 |
|  | hsa-miR-4284 | 0,60988918 | 2,11E-04 | 1,27E-03 |
|  | hsa-miR-106b-5p | 0,82944936 | 2,13E-04 | 1,27E-03 |
|  | hsa-miR-1273e | 0,63067662 | 2,75E-04 | 1,60E-03 |
|  | hsa-miR-622 | 0,63586832 | 3,24E-04 | 1,86E-03 |
|  | hsa-miR-877-3p | -0,5781655 | 3,53E-04 | 1,99E-03 |
|  | hsa-miR-1825 | -0,8566416 | 4,08E-04 | 2,26E-03 |
|  | hsa-miR-483-5p | 0,63806749 | 4,37E-04 | 2,38E-03 |
|  | kshv-miR-K12-5* | 0,57105905 | 4,45E-04 | 2,38E-03 |
|  | hsa-miR-1274b_v16.0 | -0,6000207 | 4,88E-04 | 2,57E-03 |
|  | hsa-miR-765 | 0,51110139 | 6,13E-04 | 3,14E-03 |
|  | hsa-miR-125a-5p | -0,6265232 | 6,16E-04 | 3,14E-03 |
|  | hsa-miR-100-5p | -0,8581824 | 6,26E-04 | 3,14E-03 |
|  | hsa-miR-193a-5p | -0,46089 | 7,44E-04 | 3,67E-03 |
|  | hsa-miR-125a-3p | 0,36111173 | 8,17E-04 | 3,98E-03 |
|  | hsa-miR-28-5p | -0,6793453 | 8,78E-04 | 4,21E-03 |
|  | hsa-miR-3156-5p | 0,57768219 | 9,56E-04 | 4,51E-03 |
|  | hsa-miR-501-5p | 0,29796413 | 1,08E-03 | 5,00E-03 |
|  | hsa-miR-30a-5p | -0,7528979 | 1,11E-03 | 5,04E-03 |
|  | hsa-miR-1281 | -0,7601248 | 1,12E-03 | 5,04E-03 |
|  | hsa-miR-25-3p | 0,58161406 | 1,21E-03 | 5,41E-03 |
|  | hsa-miR-146a-5p | 0,54119911 | 1,23E-03 | 5,42E-03 |
|  | hsa-miR-194-3p | 0,21314568 | 1,51E-03 | 6,54E-03 |
|  | hsa-miR-3934 | 0,43734049 | 1,62E-03 | 6,95E-03 |
|  | hsa-miR-125b-5p | -0,8449698 | 1,82E-03 | 7,68E-03 |
|  | hsa-miR-21-3p | 0,58285164 | 2,09E-03 | 8,72E-03 |
|  | hsa-miR-514b-5p | 0,37778724 | 2,30E-03 | 9,47E-03 |
|  | hsa-miR-371a-5p | 0,45857366 | 2,41E-03 | 9,81E-03 |
|  | hsa-miR-151a-3p | 0,37122413 | 2,45E-03 | 9,82E-03 |
|  | hsa-miR-4299 | 0,42647189 | 2,48E-03 | 9,82E-03 |
|  | hsa-miR-3198 | 0,5694316 | 2,67E-03 | 1,05E-02 |
|  | hsa-miR-892b | 0,32236508 | 2,74E-03 | 1,06E-02 |
|  | hsa-miR-30c-5p | -0,5709735 | 2,95E-03 | 1,12E-02 |
|  | hsa-miR-617 | 0,34973524 | 2,96E-03 | 1,12E-02 |
|  | hsa-miR-345-5p | 0,31834636 | 3,68E-03 | 1,38E-02 |
|  | hsa-miR-29a-3p | 0,65085436 | 3,95E-03 | 1,46E-02 |
|  | hsa-miR-1274a_v16.0 | -0,6105048 | 4,08E-03 | 1,49E-02 |
|  | hsa-miR-513a-5p | 0,53220884 | 4,14E-03 | 1,49E-02 |
|  | hsa-miR-3646 | -0,3289688 | 4,20E-03 | 1,50E-02 |
|  | hsa-miR-1280 | -0,4568893 | 4,36E-03 | 1,53E-02 |
|  | hsa-miR-375 | 0,5870943 | 4,38E-03 | 1,53E-02 |
|  | hsa-miR-574-5p | -0,5258208 | 4,57E-03 | 1,58E-02 |
|  | hsa-miR-3202 | 0,40975378 | 4,80E-03 | 1,64E-02 |
|  | hsa-miR-1181 | 0,39604356 | 5,46E-03 | 1,84E-02 |
|  | hsa-miR-3195 | -0,3534892 | 5,84E-03 | 1,95E-02 |
|  | hsa-miR-3622b-5p | 0,35039945 | 5,94E-03 | 1,96E-02 |
|  | hsa-miR-22-3p | -0,5594921 | 5,99E-03 | 1,96E-02 |
|  | hsa-miR-4306 | 0,3872522 | 6,17E-03 | 2,00E-02 |
|  | hsa-miR-3132 | 0,34562033 | 6,39E-03 | 2,05E-02 |
|  | hsa-miR-1288 | 0,42586448 | 6,65E-03 | 2,11E-02 |
|  | hsa-miR-3125 | 0,48341626 | 7,15E-03 | 2,25E-02 |
|  | hsa-miR-149-5p | -0,5186715 | 7,27E-03 | 2,27E-02 |
|  | hsa-miR-214-3p | -0,618092 | 7,64E-03 | 2,36E-02 |
|  | hsa-miR-3692-5p | 0,35980468 | 7,86E-03 | 2,40E-02 |
|  | hsa-miR-1225-5p | 0,25302243 | 8,18E-03 | 2,48E-02 |
|  | hsa-miR-1305 | 0,42968753 | 8,48E-03 | 2,53E-02 |
|  | hsa-miR-181a-5p | 0,52688825 | 8,51E-03 | 2,53E-02 |
|  | hsa-miR-4323 | -0,2765802 | 8,64E-03 | 2,54E-02 |
|  | hsa-miR-150-5p | -0,6155731 | 8,71E-03 | 2,54E-02 |
|  | hsa-miR-3147 | 0,24731849 | 9,68E-03 | 2,77E-02 |
|  | hsa-miR-760 | 0,30644162 | 9,72E-03 | 2,77E-02 |
|  | hsv1-miR-H6-3p | -0,562348 | 9,75E-03 | 2,77E-02 |
|  | hsa-miR-342-3p | -0,4853416 | 1,10E-02 | 3,10E-02 |
|  | hsa-miR-4257 | 0,40102439 | 1,12E-02 | 3,13E-02 |
|  | hsa-let-7e-5p | -0,6073244 | 1,13E-02 | 3,14E-02 |
|  | hsa-miR-1268a | 0,26857158 | 1,16E-02 | 3,18E-02 |
|  | hsa-miR-2276 | 0,39230797 | 1,19E-02 | 3,23E-02 |
|  | hsa-miR-513b | 0,40353173 | 1,20E-02 | 3,24E-02 |
|  | hsa-miR-130a-3p | -0,619278 | 1,29E-02 | 3,46E-02 |
|  | hbv-miR-B2RC | 0,36482673 | 1,32E-02 | 3,48E-02 |
|  | kshv-miR-K12-7* | 0,43758893 | 1,32E-02 | 3,48E-02 |
|  | hsa-miR-135a-3p | 0,44540427 | 1,51E-02 | 3,89E-02 |
|  | hsa-miR-936 | 0,28420345 | 1,51E-02 | 3,89E-02 |
|  | hsa-miR-484 | -0,2018072 | 1,51E-02 | 3,89E-02 |
|  | ebv-miR-BART19-3p | 0,47299925 | 1,55E-02 | 3,96E-02 |
|  | hsa-miR-3127-5p | 0,32974723 | 1,60E-02 | 4,05E-02 |
|  | hsa-miR-887 | 0,36932201 | 1,62E-02 | 4,07E-02 |
|  | hsa-miR-3141 | 0,31184381 | 1,77E-02 | 4,39E-02 |
|  | hsa-miR-21-5p | 0,61990861 | 1,78E-02 | 4,39E-02 |
|  | hsa-miR-1914-3p | 0,38403477 | 1,82E-02 | 4,45E-02 |
|  | hsa-miR-3197 | 0,2796403 | 1,87E-02 | 4,55E-02 |
|  | hsa-miR-193b-3p | -0,3476182 | 1,93E-02 | 4,66E-02 |
|  | hsa-miR-516a-5p | 0,20627556 | 2,01E-02 | 4,82E-02 |

**In the authors’ opinion, Table 3 (requested by the referee) could be better located in supplementary material as “Supplementary Table 2”**

| **Supplementary Table 2: Number of validated targets, pathway enrichment of validated target and known association of differentially expressed miRNAs to colorectal cancer** | | | |
| --- | --- | --- | --- |
| **ID** | **target** | **Pathway enrichment**  **of validated target** | **Mir2Disease**  **PMID Brief Description from the abstract** |
| miR-574-3p | none | none | \| 22490519 \| miR-574-5p negatively regulates Qki6/7 to impact β-catenin/Wnt signalling and the development of colorectal cancer. \| \| --- \| --- \| |
| miR-145-5p | 88 | \| hsa05219:Bladder cancer \| \| --- \| \| hsa05200:Pathways in cancer \| \| hsa05220:Chronic myeloid leukemia \| \| hsa05214:Glioma \| \| hsa04115:p53 signaling pathway \| \| hsa05218:Melanoma \| | \| 16847880 \| reduced \| \| --- \| --- \| \| 19287964 \| miR-145: down-regulated \| \| 19242066 \| miR-145: down-regulated in both colon and rectal cancer \| \| 19047896 \| miR-145: increased expression \| \| 21690566 \| The authors' findings show that chemically unmodified miRNAs complexed with PEI can be used in an efficient and biocompatible strategy of miRNA replacement therapy, as illustrated by efficacious delivery of PEI/miR-145 and PEI/miR-33a complexes in colon carcinoma. \| \| 21722265 \| The miRNA has a role in colorectal liver metastases. \| \| 21826996 \| downregulated in colon cancer compared to normal colonic mucosa \| \| 22273643 \| Down-regulation of fecal miR-143 and miR-145 are potential markers for colorectal cancer. \| \| 23128394 \| Analysis of the combined action of miR-143 and miR-145 on oncogenic pathways in colorectal cancer cells reveals a coordinate program of gene repression \| \| 23397547 \| Downregulation of anti-oncomirs miR-143/145 cluster occurs before APC gene aberration in the development of colorectal tumors \| \| 23499891 \| Tumor-suppressive microRNA-145 targets catenin to regulate Wnt/beta-catenin signaling in human colon cancer cells \| |
| miR-133b | 19 | \| hsa05218:Melanoma \| \| --- \| \| hsa04520:Adherens junction \| | \| 21573504 \| High expression of miR-185 and low expression of miR-133b were correlated with poor survival (p=0.001 and 0.028, respectively) and metastasis (p=0.007 and 0.036, respectively) in colorectal cancer. \| \| --- \| --- \| |
| miR-143-3p | 16 | \| hsa05215:Prostate cancer \| \| --- \| \| hsa04722:Neurotrophin signaling p. \| \| hsa05223:Non-small cell lung cancer \| \| hsa05214:Glioma \| \| hsa05218:Melanoma \| \| hsa05220:Chronic myeloid leukemia \| \| hsa04910:Insulin signaling pathway \| \| hsa05200:Pathways in cancer \| \| hsa05219:Bladder cancer \| \| hsa05213:Endometrial cancer \| \| hsa05221:Acute myeloid leukemia \| \| hsa04510:Focal adhesion \| \| hsa05211:Renal cell carcinoma \| \| hsa04662:B cell receptor signaling p. \| \| hsa04370:VEGF signaling pathway \| \| hsa04664:Fc epsilon RI signaling p. \| \| hsa05210:Colorectal cancer \| \| hsa05222:Small cell lung cancer \| \| hsa04012:ErbB signaling path \| \| hsa04540:Gap junction \| \| hsa04010:MAPK signaling path \| \| hsa04912:GnRH signaling path \| \| hsa04660:T cell receptor signaling p. \| | \| 16847880 \| reduced \| \| --- \| --- \| \| 19287964 \| miR-143: down-regualted \| \| 19242066 \| miR-143: only down-regulated in colon cancer but not in rectal cancer \| \| 20094072 \| miR-143:miR-143 and -145 are important onco-related genes for the initiation step of colorectal tumor development \| \| 20620599 \| miR-143:MiR-21 and miR-143 expressions were quantified by using the quantitative reverse transcription polymerase chain reaction method \| \| 21722265 \| The miRNA has a role in colorectal liver metastases. \| \| 21826996 \| downregulated in colon cancer compared to normal colonic mucosa \| \| 22241525 \| Six miRNAs were up-regulated from non-neoplastic tissue to dysplasia, but down-regulated from dysplasia to cancer (miR-122, miR-181a, miR-146b-5p, let-7e, miR-17, miR-143) \| \| 22273643 \| Down-regulation of fecal miR-143 and miR-145 are potential markers for colorectal cancer. \| \| 22533346 \| MicroRNA-143 targets MACC1 to inhibit cell invasion and migration in colorectal cancer. \| \| 22549179 \| Down-regulation of KRAS-interacting miRNA-143 predicts poor prognosis but not response to EGFR-targeted agents in colorectal cancer. \| \| 23128394 \| Analysis of the combined action of miR-143 and miR-145 on oncogenic pathways in colorectal cancer cells reveals a coordinate program of gene repression \| \| 23574723 \| MicroRNA-143 inhibits tumor growth and angiogenesis and sensitizes chemosensitivity to oxaliplatin in colorectal cancers \| \| 19638978 \| MicroRNA-143 targets DNA methyltransferases 3A in colorectal cancer. \| \| 19137007 \| miR-143 directly recognize the 3'-untranslated region of KRAS transcripts \| \|  \|  \| |
| miR-1 | 49 | none |  |
| miR-195-5p | 18 | \| hsa05212:Pancreatic cancer \| \| --- \| \| hsa05222:Small cell lung cancer \| \| hsa05200:Pathways in cancer \| \| hsa04110:Cell cycle \| \| hsa04115:p53 signaling pathway \| \| hsa05220:Chronic myeloid leukemia \| \| hsa05215:Prostate cancer \| \| hsa05219:Bladder cancer \| \| hsa05223:Non-small cell lung cancer \| \| hsa05214:Glioma \| \| hsa05218:Melanoma \| \| hsa04510:Focal adhesion \| | \| 20727858 \| miR-195:microRNA-195 promotes apoptosis and suppresses tumorigenicity of human colorectal cancer cells \| \| --- \| --- \| \| 21390519 \| Downregulation of miR-195 correlates with lymph node metastasis and poor prognosis in colorectal cancer. \| \| 22479426 \| The driving miRNAs were miR-195, miR-1280, miR-140-3p and miR-1246 in colorectal tumors. \| \| 23526568 \| MicroRNA-195 chemosensitizes colon cancer cells to the chemotherapeutic drug doxorubicin by targeting the first binding site of BCL2L2 mRNA \| |
| miR-143-5p | none | none | \| 16847880 \| reduced \| \| --- \| --- \| \| 19287964 \| miR-143: down-regualted \| \| 19242066 \| miR-143: only down-regulated in colon cancer but not in rectal cancer \| \| 20094072 \| miR-143:miR-143 and -145 are important onco-related genes for the initiation step of colorectal tumor development \| \| 20620599 \| miR-143:MiR-21 and miR-143 expressions were quantified by using the quantitative reverse transcription polymerase chain reaction method \| \| 21722265 \| The miRNA has a role in colorectal liver metastases. \| \| 21826996 \| downregulated in colon cancer compared to normal colonic mucosa \| \| 22241525 \| Six miRNAs were up-regulated from non-neoplastic tissue to dysplasia, but down-regulated from dysplasia to cancer (miR-122, miR-181a, miR-146b-5p, let-7e, miR-17, miR-143) \| \| 22273643 \| Down-regulation of fecal miR-143 and miR-145 are potential markers for colorectal cancer. \| \| 22533346 \| MicroRNA-143 targets MACC1 to inhibit cell invasion and migration in colorectal cancer. \| \| 22549179 \| Down-regulation of KRAS-interacting miRNA-143 predicts poor prognosis but not response to EGFR-targeted agents in colorectal cancer. \| \| 23128394 \| Analysis of the combined action of miR-143 and miR-145 on oncogenic pathways in colorectal cancer cells reveals a coordinate program of gene repression \| \| 23574723 \| MicroRNA-143 inhibits tumor growth and angiogenesis and sensitizes chemosensitivity to oxaliplatin in colorectal cancers \| \| 19638978 \| MicroRNA-143 targets DNA methyltransferases 3A in colorectal cancer. \| \| 19137007 \| miR-143 directly recognize the 3'-untranslated region of KRAS transcripts \| \|  \|  \| |
| miR-1260a | none | none | none |
| miR-197-3p | 2 | none | none |
| miR-1260b | none | none | none |
|  |  |  |  |
| miR-200b-3p | 32 | \| hsa05200:Pathways in cancer \| \| --- \| \| hsa05215:Prostate cancer \| \| hsa04510:Focal adhesion \| \| hsa05222:Small cell lung cancer \| | \| 22286765 \| SIX1-induced CDH1 repression and EMT in CRC cells were correlated at least in part with posttranscriptional ZEB1 activation and miR-200-family transcriptional repression. \| \| --- \| --- \| \| 22804917 \| In KRAS mutated tumours increased miR-200b and decreased miR-143 expression were associated with a good progression-free survival . \| \| 23441132 \| Down-Regulation of the miRNA-200 Family at the Invasive Front of Colorectal Cancers with Degraded Basement Membrane Indicates EMT Is Involved in Cancer Progression \| |
| miR-141-3p | 36 | none | \| 19830559 \| miR-141:miR-141 regulates SIP1 to inhibit migration and invasion of CRC cells \| \| --- \| --- \| \| 21445232 \| High levels of plasma miR-141 predicted poor survival in both cohorts and that miR-141 was an independent prognostic factor for advanced colon cancer. \| |
| miR-200c-3p | 42 | \| hsa04510:Focal adhesion \| \| --- \| \| hsa04722:Neurotrophin signaling p. \| \| hsa05200:Pathways in cancer \| \| hsa05222:Small cell lung cancer \| \| hsa05215:Prostate cancer \| | \| 21826996 \| upregulated in colon cancer compared to normal colonic mucosa \| \| --- \| --- \| \| 21873159 \| Oncogenic KRAS Regulates miR-200c and miR-221/222 in a 3D-Specific Manner in Colorectal Cancer Cells. \| \| 22286765 \| SIX1-induced CDH1 repression and EMT in CRC cells were correlated at least in part with posttranscriptional ZEB1 activation and miR-200-family transcriptional repression. \| \| 22407310 \| miR-200c inhibits invasion and migration in human colon cancer cells SW480/620 by targeting ZEB1.miR-200c inhibits metastatic ability by targeting ZEB1 in colon cancer cells SW480/620 and suggested that modulation of miR-200c could serve as therapeutic tool for inhibiting metastasis in colorectal cancer. \| \| 22641662 \| KRAS up-regulates the expression of miR-181a, miR-200c and miR-210 in a three-dimensional-specific manner in DLD-1 colorectal cancer cells. \| \| 22735571 \| MicroRNA-200c modulates epithelial-to-mesenchymal transition (EMT) in human colorectal cancer metastasis. \| \| 23441132 \| Down-Regulation of the miRNA-200 Family at the Invasive Front of Colorectal Cancers with Degraded Basement Membrane Indicates EMT Is Involved in Cancer Progression \| |
| miR-192-5p | 25 | hsa04110:Cell cycle |  |
| miR-194-5p | 13 | none | \| 21722265 \| The miRNA has a role in colorectal liver metastases. \| \| --- \| --- \| \| 21722265 \| The miRNA has a role in colorectal liver metastases. \| \| 22028325 \| p53-responsive miR-194 inhibits thrombospondin-1 and promotes angiogenesis in colon cancers. \| \| 22028325 \| p53-responsive miR-194 inhibits thrombospondin-1 and promotes angiogenesis in colon cancers. \| |
| miR-200a-3p | 29 | \| hsa04520:Adherens junction \| \| --- \| \| hsa05210:Colorectal cancer \| \| hsa05200:Pathways in cancer \| \| hsa04310:Wnt signaling pathway \| | \| 21327300 \| Compared with the respective normal tissues, the predominant alteration in tumor tissues was increased methylation for the miRNAs 1-1, 124a-1, 124a-2, 124a-3, 148a, 152, and 18b; decreased methylation for 200a and 208a; \| \| --- \| --- \| \| 22286765 \| SIX1-induced CDH1 repression and EMT in CRC cells were correlated at least in part with posttranscriptional ZEB1 activation and miR-200-family transcriptional repression. \| \| 23441132 \| Down-Regulation of the miRNA-200 Family at the Invasive Front of Colorectal Cancers with Degraded Basement Membrane Indicates EMT Is Involved in Cancer Progression \| |
| miR-429 | 24 | none | \| 23111103 \| MiR-429 is an independent prognostic factor in colorectal cancer and exerts its anti-apoptotic function by targeting SOX2 \| \| --- \| --- \| |
| miR-215 | none | none | \| 21532750 \| miR-215, miR-137, miR-708, miR-31,and miR-135b were differentially expressed in APC tumors and miR-215, miR-133a,miR-467d, miR-218, miR-708, miR-31, and miR-135b in colitis-associated tumors. \| \| --- \| --- \| \| 21752725 \| miR-215 has a unique potential as a prognostic biomarker in stage II and III colon cancer. \| \| 22469014 \| MiR-215, miR-375, miR-378 and miR-422a were significantly decreased, whereas miR-135b was increased in CRC tumor tissues. Levels of miR-215 and miR-422a correlated with clinical stage. \| \| 23532818 \| MicroRNA-215 inhibits relapse of colorectal cancer patients following radical surgery \| |
| miR-3651 | none | none |  |
| miR-210 | none | none | \| 22641662 \| KRAS up-regulates the expression of miR-181a, miR-200c and miR-210 in a three-dimensional-specific manner in DLD-1 colorectal cancer cells. \| \| --- \| --- \| |
| miR-20a-5p | 28 | \| hsa05200:Pathways in cancer \| \| --- \| \| hsa05219:Bladder cancer \| \| hsa05220:Chronic myeloid leukemia \| \| hsa04110:Cell cycle \| \| hsa05215:Prostate cancer \| \| hsa05214:Glioma \| \| hsa05218:Melanoma \| \| hsa05212:Pancreatic cancer \| \| hsa05222:Small cell lung cancer \| \| hsa05223:Non-small cell lung cancer \| \| hsa05210:Colorectal cancer \| \| hsa04350:TGF-beta signaling path \| \| hsa05213:Endometrial cancer \| \| hsa04510:Focal adhesion \| \| hsa05221:Acute myeloid leukemia \| \| hsa04115:p53 signaling pathway \| \| hsa05211:Renal cell carcinoma \| \| hsa04012:ErbB signaling path \| \| hsa05216:Thyroid cancer \| | \| 19287964 \| miR-20a: up-regulated \| \| --- \| --- \| \| 19047896 \| miR-20a: increased expression \| \| 21242194 \| miR-20a targets BNIP2 and contributes chemotherapeutic resistance in colorectal adenocarcinoma SW480 and SW620 cell lines. \| \| 21826996 \| upregulated in colon cancer compared to normal colonic mucosa \| \| 22202009 \| miR-20a, miR-21, miR-106a, miR-181b, miR-203, and miR-324-5p were stable in colorectal cancer archival tissue blocks. \| \| 22308110 \| Histone deacetylase inhibition in colorectal cancer cells reveals competing roles for members of the oncogenic miR-17-92 cluster. \| |
| miR-1246 | none | none | \| 22479426 \| The driving miRNAs were miR-195, miR-1280, miR-140-3p and miR-1246 in colorectal tumors. \| \| --- \| --- \| |
| miR-17-5p | 43 | \| hsa05200:Pathways in cancer \| \| --- \| \| hsa04110:Cell cycle \| \| hsa05219:Bladder cancer \| \| hsa05212:Pancreatic cancer \| \| hsa05220:Chronic myeloid leukemia \| \| hsa05222:Small cell lung cancer \| \| hsa05215:Prostate cancer \| \| hsa05214:Glioma \| \| hsa05218:Melanoma \| \| hsa05210:Colorectal cancer \| \| hsa04350:TGF-beta signaling path \| \| hsa05223:Non-small cell lung cancer \| \| hsa04510:Focal adhesion \| \| hsa04115:p53 signaling pathway \| \| hsa04310:Wnt signaling pathway \| \| hsa05213:Endometrial cancer \| | \| 19201770 \| miR-17-3p: up-regulated both in plasma and tissue samples \| \| --- \| --- \| \| 19287964 \| miR-17-5p: up-regulated \| \| 21826996 \| upregulated in colon cancer compared to normal colonic mucosa \| \| 22132820 \| Up-regulated miR-17 promotes cell proliferation, tumor growth and cell cycle progression by targeting RND3 tumor suppressor gene in colorectal carcinoma. \| \| 22241525 \| Six miRNAs were up-regulated from non-neoplastic tissue to dysplasia, but down-regulated from dysplasia to cancer (miR-122, miR-181a, miR-146b-5p, let-7e, miR-17, miR-143) \| \| 22308110 \| Histone deacetylase inhibition in colorectal cancer cells reveals competing roles for members of the oncogenic miR-17-92 cluster. \| \| 23250421 \| Elevated oncofoetal miR-17-5p expression regulates colorectal cancer progression by repressing its target gene P130 \| |
| miR-92a-3p | 16 | none | \| 19876917 \| miR-92a:plasma miR-29a and miR-92a have significant diagnostic value for advanced neoplasia \| \| --- \| --- \| \| 21826996 \| upregulated in colon cancer compared to normal colonic mucosa \| \| 21922590 \| The presence of the KRAS mutation was associated with up-regulation of miR-127-3p, miR-92a, and miR-486-3p and down-regulation of miR-378. \| \| 21930727 \| The expression of miR-21 and miR-92a was significantly higher in CRC tissues compared with their adjacent normal tissues \| \| 22308110 \| Histone deacetylase inhibition in colorectal cancer cells reveals competing roles for members of the oncogenic miR-17-92 cluster. \| \| 22772712 \| Overexpression of miR-92a correlates with tumor metastasis and poor prognosis in patients with colorectal cancer. \| |
| miR-1973 | none | none | none |
| miR-19b-3p | 17 | none | \| 21406606 \| Diet and carcinogen exposure modulated a number of microRNAs (miR-16, miR-19b, miR-21, miR26b, miR27b, miR-93 and miR-203) linked to canonical oncogenic signaling pathways. \| \| --- \| --- \| \| 21722265 \| The miRNA has a role in colorectal liver metastases. \| \| 22308110 \| Histone deacetylase inhibition in colorectal cancer cells reveals competing roles for members of the oncogenic miR-17-92 cluster. \| \|  \|  \| \|  \|  \| |

**Supplementary Table 3. RT-PCR and microarray analysis of the 13 DE miRNA selected in tumor and matched stroma samples.** All data are median distribution (IQ-range:2.5-97.5 percentile) of fluorescence intensity, normalized as described in the Methods section. R, the ratio of the median distribution of tumor to stroma samples measured by RT-PCR; P, the level of significance according to the Wilcoxon Rank test. Ra, the fold change (ratio) between tumor and stromal samples from the array analysis (in natural scale); q, the corrected p-value (q-value) from the array analysis.

|  | **Real Time PCR** | | | | **Array** | |
| --- | --- | --- | --- | --- | --- | --- |
| **Subtypes**  **miRNA** | **Stroma (N=51)**  **(IQ-range)** | **Tumor (N=51)**  **(IQ-range)** | **R** | ***p*** | **Ra** | ***q (%)*** |
| **miR-200c-3p** | 1·43  (*0·7-3·1)* | 7·20  (*4·3-9·9)* | 5·01 | *<0·0001* | 4.41 | < 0.0001 |
| **miR-141-3p** | 0·14  (*0·07-0·27)* | 0·34  (0·23*-*0·53*)* | 2·36 | *<0·0001* | 4.66 | < 0.0001 |
| **miR-200b-3p** | 0·73  (*0·16-1·39)* | 2·58  (*1·38-4·71)* | 3·51 | *<0·0001* | 5.45 | < 0.0001 |
| **miR-200a-3p** | 0·07  (*0·03-0·15)* | 0·18  (*0·09-0·34)* | 2·47 | *<0·0001* | 2.68 | < 0.0001 |
| **miR-1246** | 0·96  (*0·51-2·23)* | 2·84  (*1·11-5·86)* | 2·94 | *<0·0001* | 1.13 | < 0.0001 |
| **miR-92a-3p** | 0·36  (*0·28-0·61)* | 0·70  (*0·44-1·04)* | 1·92 | *<0·0001* | 1.12 | < 0.0001 |
| **miR-194-5p** | 0·24  (*0·05-0·59)* | 0·57  (*0·34-1·19)* | 2·34 | *<0·0001* | 3.0 | < 0.0001 |
| **miR-192-5p** | 0·29  (*0·07-0·51)* | 0·53  (*0·37-1·38)* | 1·79 | *<0·0001* | 3.12 | < 0.0001 |
| **miR-3651** | 0·83  (*0·57-1·25)* | 2·54  (*1·38-3·10)* | 3·05 | *<0·0001* | 1.62 | < 0.0001 |
| **miR-574-3p** | 0·45  (*0·32-0·69)* | 0·30  (*0·23-0·45)* | 0·68 | *0·0074* | 0.26 | < 0.0001 |
| **miR-197-3p** | 0·55  (*0·42-0·76)* | 0·53  (*0·38-0·89)* | 0·97 | *0·5189* | 0.45 | < 0.0001 |
| **miR-1260a** | 0·01  (*0·01-0·02)* | 0·01  (*0·02-0·02)* | 0·84 | *0·8408* | 0.44 | < 0.0001 |
| **miR-1260b** | 0·53  (*0·41-0·75)* | 0·47  (*0·32-0·70)* | 0·89 | *0·1366* | 0.46 | < 0.0001 |
