# Supplementary material for: Analysis of Differential miRNA Expression in Primary Tumor and Stroma of Colorectal Cancer Patients
Source: Biomed Res Int. 2014 Jul 10;2014:840921. doi: 10.1155/2014/840921 (PMC4128171; doi:10.1155/2014/840921)
Supplement: Supplementary file 1 — Supplementary Figure 1. Bootstrap hierarchical clustering of normalized data passing the QC filter (321 microRNAs). Each column represents a sample (T, tumor; S, stroma). The red branches in the dendrogram indicate an approximately unbiased (AU) confidence level greater than 95%. Supplementary Figure 2. Heatmap and bootstrap hierarchical clustering of differentially expressed microRNA in tumor versus stroma comparisons. Each row represents a single microRNA, and each column a matched tumor sample (T) versus stromal sample (S) comparison. Green colors indicate down-regulation (negative log fold change), while red colors indicate up-regulation (positive fold change). The red branches in the dendrograms indicate an approximately unbiased (AU) confidence greater than 95%. Supplementary Table 1. Complete list of differentially expressed miRNA between tumor and matched stroma samples. ID, official miRNA name according to miRBASE version 19; logFC , log2 fold change of tumor expression versus stroma (negative for down-regulated miRNA and positive for up-regulated miRNA); p-value, the raw p-value from the statistical test; adjusted p-value, the p-value from the statistical test adjusted for multiple test comparisons (False Discovery Rate). Supplementary Table 2: Number of validated targets, pathway enrichment of validated target and known association of differentially expressed miRNAs to colorectal cancer. Supplementary Table 3. RT-PCR and microarray analysis of the 13 DE miRNA selected in tumor and matched stroma samples. All data are median distribution (IQ-range:2.5-97.5 percentile) of fluorescence intensity, normalized as described in the Methods section. R, the ratio of the median distribution of tumor to stroma samples measured by RT-PCR; P, the level of significance according to the Wilcoxon Rank test. Ra, the fold change (ratio) between tumor and stromal samples from the array analysis (in natural scale); q, the corrected p-value (q-value) from the array analysis. [file 840921.f1.zip › 840921.f1/840921.pdf]

SUPPLEMENTARY FIGURES AND TABLES LEGEND

Supplementary Figure 1. Bootstrap hierarchical clustering of normalized data passing the QC filter (321 microRNAs). Each column represents a sample (T, tumor; S, stroma). The red branches in the dendrogram indicate an approximately unbiased (AU) confidence level greater than 95%.

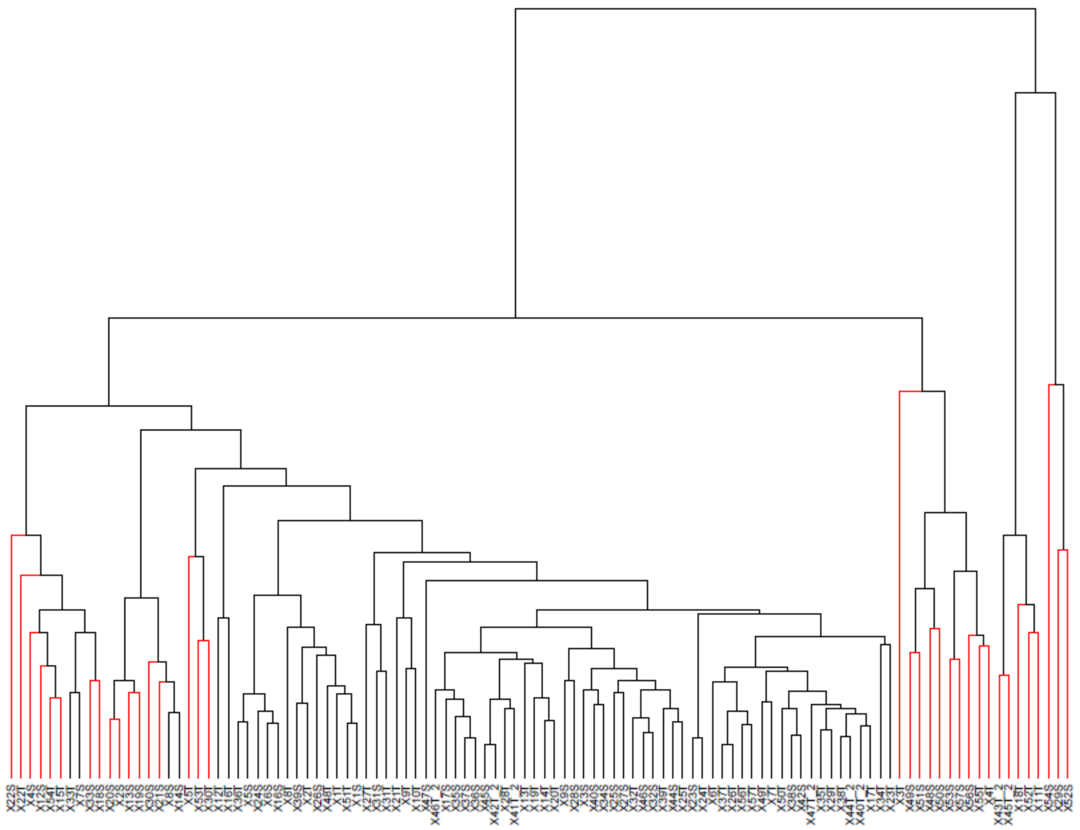

Supplementary Figure 2. Heatmap and bootstrap hierarchical clustering of differentially expressed microRNA in tumor versus stroma comparisons. Each row represents a single microRNA, and each column a matched tumor sample (T) versus stromal sample (S) comparison. Green colors indicate down-regulation (negative log fold change), while red colors indicate up-regulation (positive fold change). The red branches in the dendrograms indicate an approximately unbiased (AU) confidence greater than 95%.

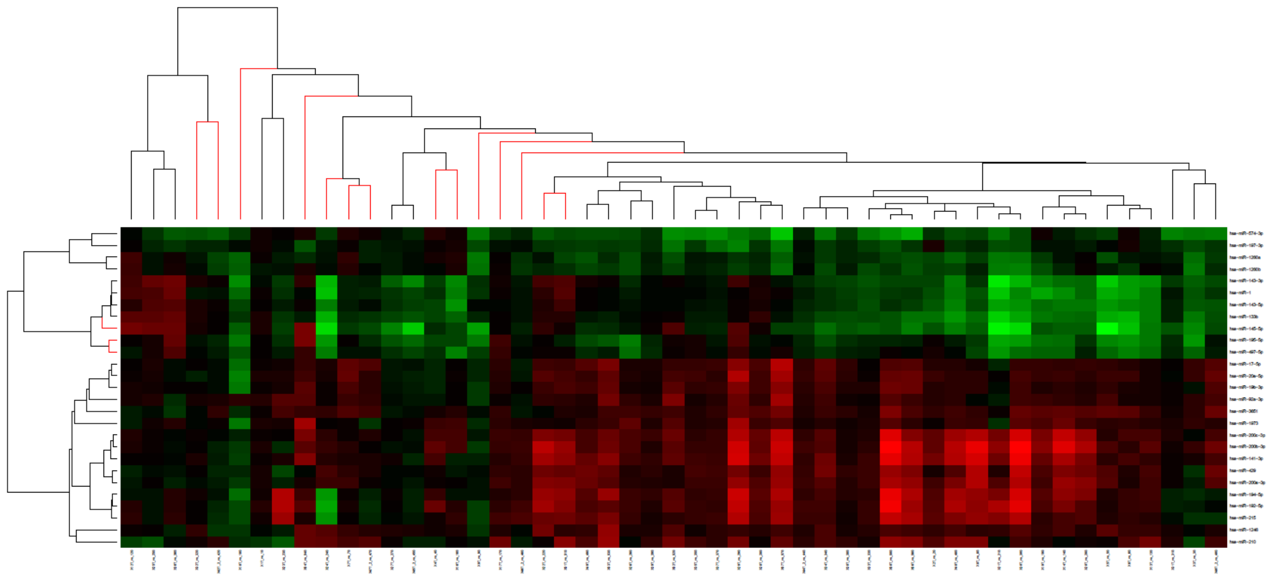

Supplementary Table 1. Complete list of differentially expressed miRNA between tumor and matched stroma samples. ID, official miRNA name according to miRBASE version 19; logFC , log2 fold change of tumor expression versus stroma (negative for down-regulated miRNA and positive for up-regulated miRNA); p-value, the raw p-value from the statistical test; adjusted p-value, the p-value from the statistical test adjusted for multiple test comparisons (False Discovery Rate).

	ID	logFC	pvalue	adjusted p-value
1.	hsa-miR-574-3p	-1,9115869	1,26E-11	4,05E-09
2.	hsa-miR-200c-3p	2,15323075	6,13E-11	9,84E-09
3.	hsa-miR-197-3p	-1,1483022	1,47E-10	1,57E-08
4.	hsa-miR-3651	1,28775706	3,74E-10	3,00E-08
5.	hsa-miR-141-3p	2,22388859	1,44E-09	8,10E-08
6.	hsa-miR-200b-3p	2,40063273	1,51E-09	8,10E-08
7.	hsa-miR-663b	0,77767795	2,39E-09	1,10E-07
8.	hsa-miR-1260a	-1,1570189	5,46E-09	2,19E-07
9.	hsa-miR-200a-3p	1,67832962	1,05E-08	3,74E-07
10.	hsa-miR-1246	1,08433315	1,38E-08	4,09E-07
11.	hsa-miR-1290	0,92604392	1,40E-08	4,09E-07
12.	hsa-miR-1260b	-1,1083897	2,35E-08	6,27E-07
13.	hsa-miR-92a-3p	1,06291954	4,64E-08	1,15E-06
14.	hsa-miR-429	1,51819183	5,59E-08	1,28E-06
15.	hsa-miR-720	-0,7269849	7,03E-08	1,50E-06
16.	hsa-miR-766-3p	-0,8696977	4,83E-07	9,70E-06
17.	hsa-miR-192-5p	1,83444059	5,25E-07	9,91E-06
18.	hsa-miR-663a	0,80827814	8,06E-07	1,44E-05
19.	hsa-miR-20a-5p	1,14909454	8,80E-07	1,45E-05
20.	hsa-miR-3654	0,65584834	9,18E-07	1,45E-05
21.	hsa-miR-203	0,89749749	9,50E-07	1,45E-05
22.	hsa-miR-194-5p	1,79583132	1,07E-06	1,57E-05
23.	hsa-miR-17-5p	1,07625287	1,25E-06	1,75E-05
24.	hsa-miR-424-3p	0,4557021	1,31E-06	1,75E-05
25.	hsa-miR-1973	1,04005254	1,47E-06	1,82E-05
26.	hsa-miR-19b-3p	1,00428691	1,48E-06	1,82E-05
27.	hsa-miR-425-5p	0,80064141	1,89E-06	2,25E-05
28.	hsa-miR-133b	-1,5123276	2,28E-06	2,61E-05
29.	hsa-miR-215	1,507275	2,89E-06	3,20E-05
30.	hsa-miR-195-5p	-1,3352339	4,54E-06	4,86E-05
31.	hsa-miR-664-5p	0,52094262	5,27E-06	5,32E-05
32.	hsa-miR-365a-3p	-0,8131199	5,30E-06	5,32E-05
33.	hsa-miR-143-5p	-1,2316102	6,15E-06	5,98E-05
34.	hsa-miR-494	0,63509731	7,89E-06	7,45E-05
35.	hsa-miR-1	-1,3411554	1,03E-05	9,46E-05
36.	hsa-miR-145-5p	-1,7477164	1,22E-05	1,09E-04
37.	hsa-miR-19a-3p	0,94241736	1,33E-05	1,15E-04
38.	hsa-miR-210	1,19299762	1,40E-05	1,18E-04
39.	hsa-miR-133a	-0,8510897	1,99E-05	1,63E-04
40.	hsa-miR-497-5p	-0,9887135	2,03E-05	1,63E-04
41.	hsa-miR-143-3p	-1,4333531	2,66E-05	2,08E-04

42.	hsa-miR-3648	0,50113222	2,87E-05	2,20E-04
43.	hsa-miR-4324	-0,5103669	3,05E-05	2,25E-04
44.	hsa-miR-130b-3p	0,59123284	3,08E-05	2,25E-04
45.	ebv-miR-BART16	-0,6430805	5,01E-05	3,58E-04
46.	hsa-miR-93-5p	0,8305619	6,89E-05	4,71E-04
47.	hsa-miR-99b-5p	-0,8048456	6,90E-05	4,71E-04
48.	hsa-miR-500a-5p	0,4459759	1,08E-04	7,22E-04
49.	hsa-miR-148a-3p	0,93546999	1,23E-04	8,09E-04
50.	hsa-miR-3154	0,44026642	1,45E-04	9,29E-04
51.	hsa-miR-99a-5p	-0,9752663	1,81E-04	1,14E-03
52.	hsa-miR-3907	-0,5207543	1,84E-04	1,14E-03
53.	hsa-miR-4284	0,60988918	2,11E-04	1,27E-03
54.	hsa-miR-106b-5p	0,82944936	2,13E-04	1,27E-03
55.	hsa-miR-1273e	0,63067662	2,75E-04	1,60E-03
56.	hsa-miR-622	0,63586832	3,24E-04	1,86E-03
57.	hsa-miR-877-3p	-0,5781655	3,53E-04	1,99E-03
58.	hsa-miR-1825	-0,8566416	4,08E-04	2,26E-03
59.	hsa-miR-483-5p	0,63806749	4,37E-04	2,38E-03
60.	kshv-miR-K12-5*	0,57105905	4,45E-04	2,38E-03
61.	hsa-miR-1274b_v16.0	-0,6000207	4,88E-04	2,57E-03
62.	hsa-miR-765	0,51110139	6,13E-04	3,14E-03
63.	hsa-miR-125a-5p	-0,6265232	6,16E-04	3,14E-03
64.	hsa-miR-100-5p	-0,8581824	6,26E-04	3,14E-03
65.	hsa-miR-193a-5p	-0,46089	7,44E-04	3,67E-03
66.	hsa-miR-125a-3p	0,36111173	8,17E-04	3,98E-03
67.	hsa-miR-28-5p	-0,6793453	8,78E-04	4,21E-03
68.	hsa-miR-3156-5p	0,57768219	9,56E-04	4,51E-03
69.	hsa-miR-501-5p	0,29796413	1,08E-03	5,00E-03
70.	hsa-miR-30a-5p	-0,7528979	1,11E-03	5,04E-03
71.	hsa-miR-1281	-0,7601248	1,12E-03	5,04E-03
72.	hsa-miR-25-3p	0,58161406	1,21E-03	5,41E-03
73.	hsa-miR-146a-5p	0,54119911	1,23E-03	5,42E-03
74.	hsa-miR-194-3p	0,21314568	1,51E-03	6,54E-03
75.	hsa-miR-3934	0,43734049	1,62E-03	6,95E-03
76.	hsa-miR-125b-5p	-0,8449698	1,82E-03	7,68E-03
77.	hsa-miR-21-3p	0,58285164	2,09E-03	8,72E-03
78.	hsa-miR-514b-5p	0,37778724	2,30E-03	9,47E-03
79.	hsa-miR-371a-5p	0,45857366	2,41E-03	9,81E-03
80.	hsa-miR-151a-3p	0,37122413	2,45E-03	9,82E-03
81.	hsa-miR-4299	0,42647189	2,48E-03	9,82E-03
82.	hsa-miR-3198	0,5694316	2,67E-03	1,05E-02
83.	hsa-miR-892b	0,32236508	2,74E-03	1,06E-02
84.	hsa-miR-30c-5p	-0,5709735	2,95E-03	1,12E-02
85.	hsa-miR-617	0,34973524	2,96E-03	1,12E-02
86.	hsa-miR-345-5p	0,31834636	3,68E-03	1,38E-02
87.	hsa-miR-29a-3p	0,65085436	3,95E-03	1,46E-02
88.	hsa-miR-1274a_v16.0	-0,6105048	4,08E-03	1,49E-02
89.	hsa-miR-513a-5p	0,53220884	4,14E-03	1,49E-02
90.	hsa-miR-3646	-0,3289688	4,20E-03	1,50E-02
91.	hsa-miR-1280	-0,4568893	4,36E-03	1,53E-02
92.	hsa-miR-375	0,5870943	4,38E-03	1,53E-02

93.	hsa-miR-574-5p	-0,5258208	4,57E-03	1,58E-02
94.	hsa-miR-3202	0,40975378	4,80E-03	1,64E-02
95.	hsa-miR-1181	0,39604356	5,46E-03	1,84E-02
96.	hsa-miR-3195	-0,3534892	5,84E-03	1,95E-02
97.	hsa-miR-3622b-5p	0,35039945	5,94E-03	1,96E-02
98.	hsa-miR-22-3p	-0,5594921	5,99E-03	1,96E-02
99.	hsa-miR-4306	0,3872522	6,17E-03	2,00E-02
100.	hsa-miR-3132	0,34562033	6,39E-03	2,05E-02
101.	hsa-miR-1288	0,42586448	6,65E-03	2,11E-02
102.	hsa-miR-3125	0,48341626	7,15E-03	2,25E-02
103.	hsa-miR-149-5p	-0,5186715	7,27E-03	2,27E-02
104.	hsa-miR-214-3p	-0,618092	7,64E-03	2,36E-02
105.	hsa-miR-3692-5p	0,35980468	7,86E-03	2,40E-02
106.	hsa-miR-1225-5p	0,25302243	8,18E-03	2,48E-02
107.	hsa-miR-1305	0,42968753	8,48E-03	2,53E-02
108.	hsa-miR-181a-5p	0,52688825	8,51E-03	2,53E-02
109.	hsa-miR-4323	-0,2765802	8,64E-03	2,54E-02
110.	hsa-miR-150-5p	-0,6155731	8,71E-03	2,54E-02
111.	hsa-miR-3147	0,24731849	9,68E-03	2,77E-02
112.	hsa-miR-760	0,30644162	9,72E-03	2,77E-02
113.	hsv1-miR-H6-3p	-0,562348	9,75E-03	2,77E-02
114.	hsa-miR-342-3p	-0,4853416	1,10E-02	3,10E-02
115.	hsa-miR-4257	0,40102439	1,12E-02	3,13E-02
116.	hsa-let-7e-5p	-0,6073244	1,13E-02	3,14E-02
117.	hsa-miR-1268a	0,26857158	1,16E-02	3,18E-02
118.	hsa-miR-2276	0,39230797	1,19E-02	3,23E-02
119.	hsa-miR-513b	0,40353173	1,20E-02	3,24E-02
120.	hsa-miR-130a-3p	-0,619278	1,29E-02	3,46E-02
121.	hbv-miR-B2RC	0,36482673	1,32E-02	3,48E-02
122.	kshv-miR-K12-7*	0,43758893	1,32E-02	3,48E-02
123.	hsa-miR-135a-3p	0,44540427	1,51E-02	3,89E-02
124.	hsa-miR-936	0,28420345	1,51E-02	3,89E-02
125.	hsa-miR-484	-0,2018072	1,51E-02	3,89E-02
126.	ebv-miR-BART19-3p	0,47299925	1,55E-02	3,96E-02
127.	hsa-miR-3127-5p	0,32974723	1,60E-02	4,05E-02
128.	hsa-miR-887	0,36932201	1,62E-02	4,07E-02
129.	hsa-miR-3141	0,31184381	1,77E-02	4,39E-02
130.	hsa-miR-21-5p	0,61990861	1,78E-02	4,39E-02
131.	hsa-miR-1914-3p	0,38403477	1,82E-02	4,45E-02
132.	hsa-miR-3197	0,2796403	1,87E-02	4,55E-02
133.	hsa-miR-193b-3p	-0,3476182	1,93E-02	4,66E-02
134.	hsa-miR-516a-5p	0,20627556	2,01E-02	4,82E-02

Supplementary Table 2. RT-PCR and microarray analysis of the 13 DE miRNA selected in tumor and matched stroma samples. All data are median distribution (IQ-range:2.5-97.5 percentile) of fluorescence intensity, normalized as described in the Methods section. R, the ratio of the median distribution of tumor to stroma samples measured by RT-PCR; P, the level of significance according to the Wilcoxon Rank test. Ra, the fold change (ratio) between tumor and stromal samples from the array analysis (in natural scale); q, the corrected p-value (q-value) from the array analysis.

	**Real Time PCR**				**Array**	
**Subtypes miRNA**	**Stroma (N=51) (IQ-range)**	**Tumor (N=51) (IQ-range)**	**R**	***p***	**Ra**	***q (%)***
**miR-200c-3p**	1·43 *(0·7-3·1)*	7·20 *(4·3-9·9)*	7·20 *(4·3-9·9)*	*<0·0001*	4.41	< 0.0001
**miR-141-3p**	0·14 *(0·07-0·27)*	0·34 (0·23-0·53)	0·34 (0·23-0·53)	*<0·0001*	4.66	< 0.0001
**miR-200b-3p**	0·73 *(0·16-1·39)*	2·58 *(1·38-4·71)*	2·58 *(1·38-4·71)*	*<0·0001*	5.45	< 0.0001
**miR-200a-3p**	0·07 *(0·03-0·15)*	0·18 *(0·09-0·34)*	0·18 *(0·09-0·34)*	*<0·0001*	2.68	< 0.0001
**miR-1246**	0·96 *(0·51-2·23)*	2·84 *(1·11-5·86)*	2·84 *(1·11-5·86)*	*<0·0001*	1.13	< 0.0001
**miR-92a-3p**	0·36 *(0·28-0·61)*	0·70 *(0·44-1·04)*	0·70 *(0·44-1·04)*	*<0·0001*	1.12	< 0.0001
**miR-194-5p**	0·24 *(0·05-0·59)*	0·57 *(0·34-1·19)*	0·57 *(0·34-1·19)*	*<0·0001*	3.0	< 0.0001
**miR-192-5p**	0·29 *(0·07-0·51)*	0·53 *(0·37-1·38)*	0·53 *(0·37-1·38)*	*<0·0001*	3.12	< 0.0001
**miR-3651**	0·83 *(0·57-1·25)*	2·54 *(1·38-3·10)*	2·54 *(1·38-3·10)*	*<0·0001*	1.62	< 0.0001
**miR-574-3p**	0·45 *(0·32-0·69)*	0·30 *(0·23-0·45)*	0·30 *(0·23-0·45)*	*0·0074*	0.26	< 0.0001
**miR-197-3p**	0·55 *(0·42-0·76)*	0·53 *(0·38-0·89)*	0·53 *(0·38-0·89)*	*0·5189*	0.45	< 0.0001
**miR-1260a**	0·01 *(0·01-0·02)*	0·01 *(0·02-0·02)*	0·01 *(0·02-0·02)*	*0·8408*	0.44	< 0.0001
**miR-1260b**	0·53 *(0·41-0·75)*	0·47 *(0·32-0·70)*	0·47 *(0·32-0·70)*	*0·1366*	0.46	< 0.0001
